# Evaluation of the Effectiveness of *Piper cubeba* Extract in the Amelioration of CCl_4_-Induced Liver Injuries and Oxidative Damage in the Rodent Model

**DOI:** 10.1155/2015/359358

**Published:** 2015-01-14

**Authors:** Mansour AlSaid, Ramzi Mothana, Mohammad Raish, Mohammed Al-Sohaibani, Mohammed Al-Yahya, Ajaz Ahmad, Mohammed Al-Dosari, Syed Rafatullah

**Affiliations:** ^1^Department of Pharmacognosy and Medicinal, Aromatic & Poisonous Plants Research Center (MAPPRC), College of Pharmacy, King Saud University, P.O. Box 2457, Riyadh 11451, Saudi Arabia; ^2^Department of Pharmaceutics, College of Pharmacy, King Saud University, P.O. Box 2457, Riyadh 11451, Saudi Arabia; ^3^Department of Medicine and Pathology, Gastroenterology Unit, College of Medicine, King Khalid University Hospital, King Saud University, P.O. Box 2925, Riyadh-11461, Saudi Arabia; ^4^Department of Clinical Pharmacy, College of Pharmacy, King Saud University, P.O. Box 2457, Riyadh 11451, Saudi Arabia

## Abstract

*Background*. Liver diseases still represent a major health burden worldwide. Moreover, medicinal plants have gained popularity in the treatment of several diseases including liver. Thus, the present study was to evaluate the effectiveness of * Piper cubeba* fruits in the amelioration of CCl_4_-induced liver injuries and oxidative damage in the rodent model. * Methods*. Hepatoprotective activity was assessed using various biochemical parameters like SGOT, SGPT, *γ*-GGT, ALP, total bilirubin, LDH, and total protein. Meanwhile, * in vivo* antioxidant activities as LPO, NP-SH, and CAT were measured in rat liver as well as mRNA expression of cytokines such as TNF*α*, IL-6, and IL-10 and stress related genes iNOS and HO-1 were determined by RT-PCR. The extent of liver damage was also analyzed through histopathological observations. * Results*. Treatment with PCEE significantly and dose dependently prevented drug induced increase in serum levels of hepatic enzymes. Furthermore, PCEE significantly reduced the lipid peroxidation in the liver tissue and restored activities of defense antioxidant enzymes NP-SH and CAT towards normal levels. The administration of PCEE significantly downregulated the CCl_4_-induced proinflammatory cytokines TNF*α* and IL-6 mRNA expression in dose dependent manner, while it upregulated the IL-10 and induced hepatoprotective effect by downregulating mRNA expression of iNOS and HO-1 gene.

## 1. Introduction 

A large number of crucial functions needed to regulate homeostasis such as detoxification and excretion are performed by liver; however liver diseases are still major health concerns in spite of the progress made in the field of medicine and pharmaceutical sciences. Numerous side effects are associated with synthetic drugs used in treating hepatic disorders. Consequently traditional herbal drugs, spices, fruits, vegetables, and medicinal plants have gained popularity over the past decades owing to their safety and efficacy.

The fruits of* Piper cubeba* L. (Piperaceae) are commonly known as cubeba in Arabic and tailed piper in English. They are spices and possess various medicinal properties [1]. In traditional medicine they are used as stimulants, appetizers, stomachics, and expectorants. The fruit of cubeba is also used to relive the gastric pain, enteritis, diarrhea, and anti-inflammatory agent. Furthermore, cubeba fruits are known to possess pain and inflammation reducing capacity in experimental animals [2], which is attributed to the antioxidant activity of some isolated chemical constituents [3]. The fruits are also reported to possess antibacterial, antifungal, bactericidal (*Helicobacter pylori*), and renal protective properties [4–6].* P. cubeba* is used by the traditional medicine practitioner in treating acute jaundice. In an earlier study ethanolic extract of* P. cubeba* has been shown to enhance the activity of pioglitazone and act synergistically in lowering the blood glucose level in rats [7]. There are several lines of evidence which implicated oxidative stress and inflammation in the etiology of liver diseases, cardiovascular diseases, and cancer [8, 9]. As a result, carbon tetrachloride (CCl_4_), which produces reactive free radicals when metabolized, has been widely used as a solvent for induction of hepatic damage in animal models [10]. CCl_4_ increases lipid peroxidation in hepatic cells and induces liver damage and necrosis [11]. To the best of our knowledge there was a lack of scientific reports available in support of its traditional claim of hepatoprotective potential. So far, there has been no research reported on hepatoprotective effect against carbon tetrachloride induced liver damage in rats. Hence, the present study was aimed at investigating the possible potential hepatoprotective effects of the* Piper cubeba* ethanolic extract (PCEE) against CCl_4_-induced hepatic injuries in male Wistar rats.

## 2. Material and Methods

### 2.1. Animals

Healthy male Wistar albino rats (180–200 g) were used for the study. Animals were issued from Central Animal House Facility of King Saud University and were kept in standard plastic animal cages in groups of 6 animals each with 12-hour light and dark cycle at 25 ± 2°C. The rats were fed on standard rat chow and provided water* ad libitum*. The animals were acclimatized to laboratory conditions for a week prior to experiments.

### 2.2. Plant Material and Preparation of Extract

The fruits of* Piper cubeba* were purchased from the local vegetable market in Riyadh and their identity was confirmed at the Department of Pharmacognosy, College of Pharmacy, King Saud University. The dried fruits (500 g) were coarsely powdered and macerated in 3 L of 70% ethanol for 72 h using percolation method. The solvent was then removed at 40°C under reduced pressure in a rotatory evaporator. The* Piper cubeba* ethanolic extract (PCEE) was then suspended in distilled water just before its administration to the animals.

### 2.3. Acute Toxicity

Male Wistar albino rats were divided into test groups comprising six animals in each group. The test was performed using increasing oral dose of herbal extract from 100 to 1000 mg/kg body weight. The rats were observed continuously for 1 h and then half hourly for 4 h for any gross behavioral change and general motor activities like writhing, convulsion, response to tail pinching, gnawing, pupil size, fecal output, feeding behavior, and so forth and further up to 72 h for any mortality. The extract does not cause any significant behavioral changes and no mortality was observed.

### 2.4. Experimental Procedure

Rats were randomly divided into five groups of six animals each. Group I received normal saline for 7 days and served as normal control. Group II received normal saline (1 mL/kg, p.o.) for 7 days and served as toxic control. Groups III and IV were prophylactically treated with plant extract at a dose of 250 and 500 mg/kg p.o. each, respectively. Group V served as positive control and was prophylactically treated with silymarin (10 mg/kg, p.o.) for 7 days. On the 8th day, Groups II, III, and IV and Group V were injected intraperitoneally (i.p.) with 0.4 mL/kg CCl_4_ as a 20% solution in paraffin oil, while the other two groups were given an equal amount of paraffin oil. After 24 h of CCl_4_-induced hepatotoxicity, the blood was collected from retro-orbital plexus under light ether anesthesia in tubes containing disodium EDTA. Plasma was separated by centrifugation at 2500 ×g for 10 min and was transferred to prelabeled eppendrof tubes for various biochemical parameters. Immediately, after blood withdrawal the animals were sacrificed and liver samples were collected for histopathological and biochemical estimations. Liver samples were washed with chilled normal saline, and 10% (w/v) liver homogenates were prepared in ice cold 0.15 M KCl solution using motor driven Teflon pestle. The animals were approved by Institutional Animal Ethics Committee of College of Pharmacy, King Saud University, Riyadh, Saudi Arabia.

### 2.5. Biochemical Estimation

Different biochemical parameters like serum glutamic oxaloacetic transaminase (SGOT) [12], serum glutamic pyruvic transaminase (SGPT) [13], *Υ*-glutamyl transpeptidase (GGT) [14], alkaline phosphatase [14], total bilirubin [15], and lactic acid dehydrogenase (LDH) [16], respectively, were estimated in serum.

### 2.6. Estimation of Malondialdehyde (MDA) in Hepatic Tissue

The method reported by Utely et al. [17] was followed. The animals were killed 1 h after ethanol administration. The liver tissues were removed and homogenized in 0.15 mol/L KCl (at 4°C) in a Potter-Elvehjem type C homogenizer to give a 10% w/v homogenate. Aliquots of homogenate 1 mL in volume were incubated at 37°C for 3 h in a metabolic shaker. Then 1 mL of 10% aqueous TCA was added and mixed. The mixture was then centrifuged at 800 ×g for 10 min. One milliliter of the supernatant was removed and mixed with 1 mL of 0.67% 2-thiobarbituric acid in water and placed in a boiling water bath for 10 min. The mixture was cooled and diluted with 1 mL distilled water. The resulting chromogen absorbance was determined at the wavelength of 532 nm at room temperature against blank reference. The concentration of MDA was read from standard calibration curve plotted using 1,1,3,3'-tetraethoxypropane (TEP). The extent of lipid peroxidation was expressed as MDA values are expressed as nanomoles of MDA per gram of protein using a molar extinction coefficient for MDA of 1.56 × 105 M^−1 ^cm^−1^ [18]. The protein content was estimated according to the method of Lowry et al. (1951) [19].

### 2.7. Estimation of Nonprotein Sulfhydryl (NP-SH) in Hepatic Tissue

Hepatic nonprotein sulfhydryls were measured according to the method of Sedlak and Lindsay (1968) [20]. The hepatic tissue was homogenized in ice-cold normal saline containing 0.02 mmol/L ethylenediaminetetraacetic acid (EDTA). Aliquots of 5 mL of the homogenates were mixed in 15 mL test tubes with 4 mL of distilled water and 1 mL of 50% trichloroacetic acid (TCA). The tubes were shaken intermittently for 10 min and centrifuged at 3000 rpm/min. Two milliliters of supernatant was mixed with 4 mL of 0.4 mol/L Tris buffer at pH 8.9. 0.1 mL of 5,5′-dithio-bis-(2-nitrobenzoic acid) (DTNB) was added and the sample was shaken. The absorbance was measured within 5 min of DTNB addition at 412 nm against a reagent blank.

### 2.8. Estimation of Catalase Activity and Total Protein in Hepatic Tissue

Catalase activity was measured according to the method described by Aebi (1974) [21]. Supernatant (0.1 mL) was added to cuvette containing 1.9 mL of 50 mM phosphate buffer (pH 7.0). Reaction was started by the addition of 1.0 mL of freshly prepared 30 mM H_2_O_2_. The rate of decomposition of H_2_O_2_ was measured spectrophotometrically from changes in absorbance at 240 nm. Activity of catalase was expressed as U/mg protein. The total protein was estimated in liver homogenate and determined spectrophotometrically by using Folin phenol reagent [19].

### 2.9. Total RNA Isolation

Twenty-four hours following CCl_4_ administration, the liver samples were obtained to be used for total RNA isolation according to the manufacturer's instructions of the Trizol reagent (Life Technologies, Inc., Grand Island, NY, USA). Following total RNA isolation, the reverse transcription from total RNA to cDNA was processed by high capacity cDNA reverse transcription kit of Applied Biosystems according to the manufacturer's instructions [22]. Concisely, RNA was diluted to 2 ×* RT master mix* buffer; 2 *μ*L dNTP (10 mM), 1 *μ*L RNase inhibitor, and 1 *μ*L hexamer primers were added to 10 *μ*L of diluted RNA on ice. The mixture was heated at 65°C for 10 min and then snap-cooled on ice for 2 min, prior to the addition of 2 *μ*L of reverse transcriptase (200 units *μ*L^−1^). The reaction was carried out at 25°C for 10 min then 37°C for 120 min fallowed by 72°C for 4 minutes. The cDNA was stored at −80°C until further use.

### 2.10. Expression of TNF-*α*, IL-10, HO-1, and iNOS-2 mRNA in Hepatic Tissue

Quantitative analysis of specific mRNA expression was performed by RT-PCR by subjecting the resulting cDNA to PCR amplification using 96-well optical reaction plates in the ABI Prism 7500 System (Applied Biosystems). The 25 *μ*L reaction mixture contained 0.1 *μ*L of 10 *μ*M forward primer and 0.1 *μ*L of 10 *μ*M reverse primer (40 nM final concentration of each primer), 12.5 *μ*L of SYBR Green Universal Master mix, 11.05 *μ*L of nuclease-free water, and 1.25 *μ*L of cDNA sample. Rat primers TNF-*α*, IL-10, HO-1, iNOS-2, and *β*-ACTIN gene were synthesized and purchased from Integrated DNA Technologies (IDT, Coralville, IA) (Table 1). The fold change in the level of mRNA between treated and untreated groups was corrected by the levels of *β*-ACTIN. The RT-PCR data were analyzed using the relative gene expression, that is, (DD CT) method, as described and explained previously [23, 24]. Briefly, the data are presented as the fold change in gene expression normalized to the endogenous reference gene *β*-ACTIN and relative to a calibrator. The fold change in the level of target genes between treated and untreated groups, corrected by the level of *β*-ACTIN, was determined using the following equation: fold  change = 2^−Δ(ΔCt)^, where ΔCt = Ct_(target)_ − Ct_(*β*-actin)_ and Δ(ΔCt) = ΔCt_(treated)_ − ΔCt_(untreated)_. All reactions were run in duplicate. The hot start polymerase was activated by heating at 95°C for 3 min. The cycling conditions were 0.1 min at 95°C (melting) and 0.45 min at 60°C (annealing and extension). Threshold values (Ct) were calculated automatically by the software. The Ct data was processed according to the method described by Pfaffl briefly [24]; the Pfaffl equation was first used to calculate the relative gene expression ratio, that is, the change in target gene expression divided by the change in the reference gene expression.

### 2.11. Histopathological Studies

Liver tissues were sliced in small pieces and immersed in neutral buffered 10% formalin for 24 h. The fixed tissues were processed routinely, embedded in paraffin (to get paraffin sections 4-5 *μ*m), sectioned, deparaffinized, and rehydrated using the standard techniques (Bancroft and Gamble). The sections were then stained with Haematoxylin-Eosin dye and studied for histopathological changes [25].

## 3. Studies of the* In Vitro* Antioxidant Activity

### 3.1. Scavenging Activity of DPPH Radical

The radical scavenging ability of the PCEE against DPPH was evaluated as previously described [30]. In the presence of an antioxidant which can donate an electron to DPPH, the purple color, typical for free DPPH radical decays, and the change in absorbency at *λ* = 517 nm were measured. The test provides information on the ability of a compound to donate a hydrogen atom, on the number of electrons a given molecule can donate, and on the mechanism of antioxidant action. PCEE was redissolved in methanol and various concentrations (10, 50, 100, 500, and 1000 *μ*g/mL) of the extract; 125 *μ*L prepared DPPH (1 mM in methanol) and 375 *μ*L solvent (methanol) were added. After 30 min incubation at 25°C, the decrease in absorbance was measured at *λ* = 517 nm. The radical scavenging activity was calculated from the following equation:

(1) AbssampleAbscontrol%  of  radical  scavenging  activity000=Abscontrol−AbssampleAbscontrol×100.

### 3.2. *β*-Carotene-Linoleic Acid Assay

The antioxidant activity of the extract was evaluated using the *β*-carotene bleaching method described and modified by Mothana (2011) [31]. One mL of a 0.2 mg/mL *β*-carotene solution in chloroform was added to flasks containing 0.02 mL of linoleic acid and 0.2 mL of Tween-20. The chloroform was removed at 40°C using a rotary evaporator. The resultant mixture was immediately diluted with 100 mL of distilled water and mixed for 1-2 min to form an emulsion. A mixture prepared similarly but without *β*-carotene was used as a blank. A control containing 0.2 mL of 80% (v/v) methanol instead of extract was also prepared. A 5 mL aliquot of the emulsion was added to a tube containing 0.2 mL of the sample extract at 1 mg/mL. Rutin (1 mg/mL) was used as a standard. The tubes were placed in a water bath at 40°C for 2 h. Absorbance was read at 470 nm at 15 min intervals. The antioxidant activity was calculated using the following equation:

(2) $$\begin{array}{c} \%\;\text{of}\;\text{antioxidant}\;\text{activity}=1-\frac{\left({\text{Abs}}_{0}-{\text{Abs}}_{t}\right)}{\left({\text{Abs}}_{0}^{{}^{\circ}}-{\text{Abs}}_{t}^{{}^{\circ}}\right)}\times 100, \end{array}$$

where Abs_0_ and Abs_0_° are the absorbance values measured at zero time of incubation for sample extract and control, respectively. Abs_*t*_ and Abs_*t*_° are the absorbance values for sample extract and control, respectively, at *t* = 120 min.

### 3.3. Phytochemical Screening

Preliminary phytochemical screening for terpenoids, alkaloids, flavonoids, anthraquinones, saponins, carbohydrates, tannins, and coumarins was performed with the extract by using chemical methods and thin-layer chromatography (TLC) according to the methodology described by Wagner and Bladt (1996) [32].

### 3.4. Statistical Data Analysis

Results are expressed as mean ± S.D. Total variation present in a set of data was estimated by one-way analysis of variance (ANOVA) followed by Dunnett's* t*-test. *P* < 0.01 was considered significant.

## 4. Results

### 4.1. Protective Effect of PCEE on CCl_4_-Induced Hepatotoxicity 

Preliminary studies on* Piper cubeba* extract were devoid of any toxicity in rats when given in dose up to 1000 mg/kg by oral route. Hence, for further study 250 and 500 mg/kg doses of extract were selected. A significant increase in serum biomarkers such as serum SGOT, SGPT, GGT, ALP, total bilirubin, and LDH was observed in animals treated with CCl_4_, which is indicative of hepatic failure.* PCEE* at a dose of 250 and 500 mg/kg p.o. pretreatment for 7 days decreased the levels of abovementioned parameters significantly (*P* < 0.05, *P* < 0.01, and *P* < 0.001) in groups III and IV. Moreover, silymarin (group V) pretreatment produced highly significant decrease (*P* < 0.001) in serum SGOT, SGPT, GGT, ALP total bilirubin, and LDH. The amelioration of CCl_4_-induced hepatic injuries by PCEE is comparable with silymarin (Table 2).

### 4.2. Effect of PCEE on the Level of MDA

**Figure 1:**
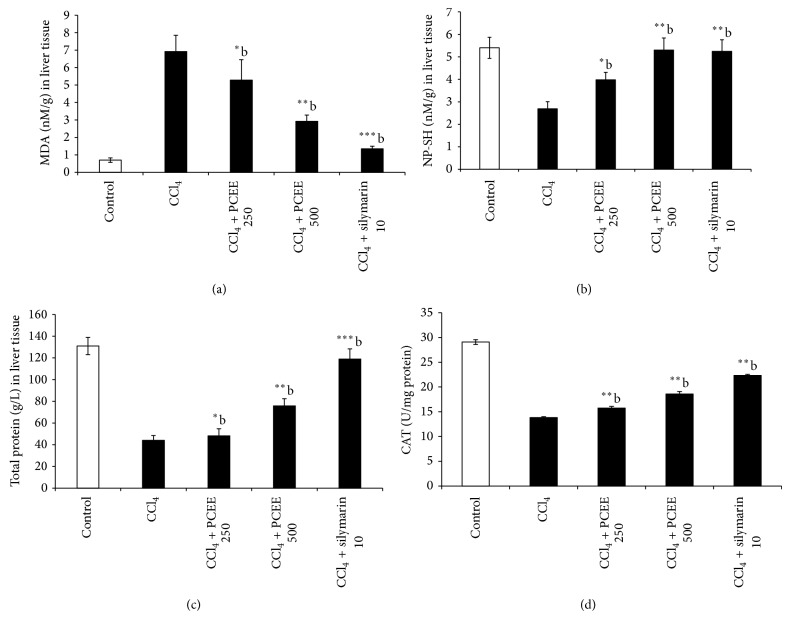
Effect of* Piper cubeba* ethanolic extract on (a) MDA, (b) NP-SH, (c) total protein level, and (d) catalase levels in the liver tissue of the rats treated with CCl_4_. All values represent mean ± SEM. ^**^ *P* < 0.01; ^***^ *P* < 0.001; ^a^ *P* < 0.05; ^b^ *P* < 0.01; ^c^ *P* < 0.001 ANOVA, followed by Dunnett's multiple comparison test. ^∗,∗∗,∗∗∗^Compared to normal group; ^a,b,c^compared to CCl_4_ only group.

The extent of lipid peroxidation is measured by the formation of thiobarbituric acid reactive substances (TBARS). There is a sharp increase in (TBARS) level in CCl_4_-treated rats (group II) indicative of oxidative stress. The PCEE 250 and 500 mg/kg + CCl_4_-treated rats (groups III and IV) showed significant dose dependent reduction of TBARS level as compared to CCl_4_-treated rats (group II). This clearly showed reduction of oxidative stress by PCEE.  Figure 1(a) showed clear significant change in the antioxidant levels of TBARS in CCl_4_ intoxicated rats as 6.92 ± 0.93 (*P* < 0.001) compared to control group. The change in TBARS level is comparable with silymarin.

### 4.3. Effect of PCEE on the Level of NP-SH

CCl_4_ intoxicated rats (group II) showed a significant decrease of NP-SH content indicative of an increase in protein metabolism whereas there was a dose dependent increase in NP-SH content significantly in groups III and IV (PCEE 250 and 500 mg/kg + CCl_4_). Silymarin pretreatment (group V) produced highly significant increase in NP-SH (*P* < 0.001). This clearly indicates that PCEE has ability to replenish the NP-SH content to normal levels (Figure 1(b)).

### 4.4. Effect of PCEE on the Level of Total Protein

Figure 1(c) showed a significant decrease of total protein content in CCl_4_ intoxicated rats which was indicative of hepatic injuries caused due to oxidative stress. Moreover, a dose dependent increase in total protein content was significantly observed in groups III and IV (PCEE 250 and 500 mg/kg + CCl_4_). Silymarin pretreatment (group V) produced highly significant increase in total proteins (*P* < 0.001). This clearly indicates that PCEE and silymarin have the ability to induce cell proliferation in hepatic tissue (Figure 1(c)).

### 4.5. Effect of PCEE on the Level of Catalase

CCl_4_ treatment caused a significant (*P* < 0.001) decrease in the level of catalase in liver tissue when compared with control group. The pretreatment with PCEE at both doses resulted in a significant increase of catalase level when compared to CCl_4_-treated rats. Silymarin showed also a significant increase in antioxidant enzymes levels compared to CCl_4_-treated rats (Figure 1(d)).

### 4.6. Effect of PCEE on mRNA Expression of Cytokines Genes Such as Tumor Necrotic Factor-*α* (TNF-*α*), Interleukin-6 (IL-6), and Interleukin-10 (IL-10)

**Figure 2:**
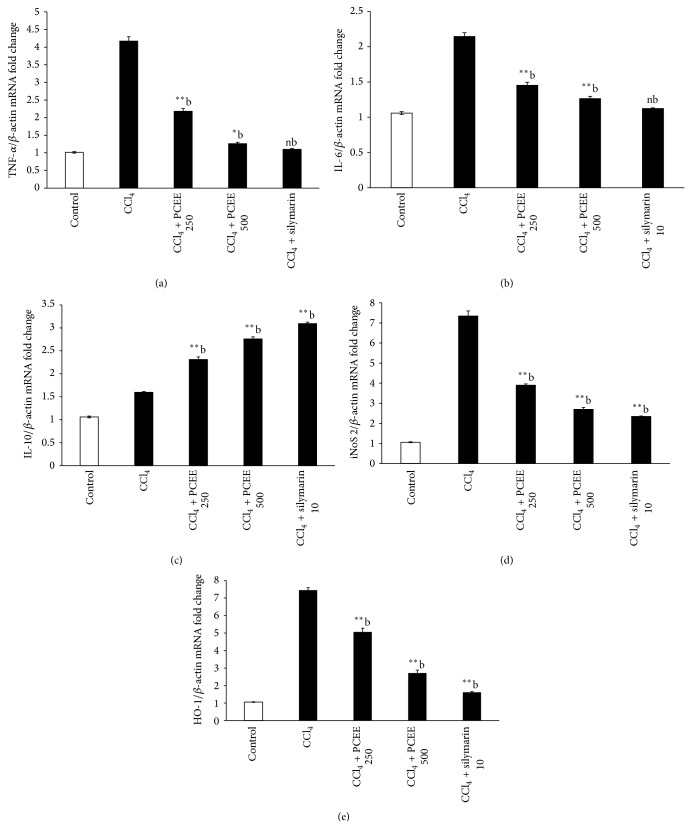
Effect of* Piper cubeba* ethanolic extract on mRNA expression of cytokines genes: (a) tumor necrotic factor-*α* (TNF-*α*), (b) interleukin-6 (IL-6), (c) interleukin-10 (IL-10), (d) inducible nitric oxide synthase gene (iNOS), and (e) inducible heamoxygenase (HO-1) gene. All values represent mean ± SEM. ^**^ *P* < 0.01; ^***^ *P* < 0.001; ^a^ *P* < 0.05; ^b^ *P* < 0.01; ^c^ *P* < 0.001 ANOVA, followed by Dunnett's multiple comparison test. ^∗,∗∗,∗∗∗^Compared to normal group; ^a,b,c^compared to CCl_4_ only group.

The hepatic level of tumor necrotic factor-*α* (TNF-*α*) and interleukin-6 (IL-6) on mRNA expression in CCl_4_ intoxicated rats was approximately 4.5- and 2.1-fold higher than that in the control rats, as shown in Figures 2(a) and 2(b), while the PCEE + CCl_4_-treated rats showed significant dose dependent inhibition of TNF-*α* and mRNA expression as compared to CCl_4_ intoxicated rats. In addition, treatment of rats with PCEE for one week prior to CCl_4_ administration increased the mRNA expression of IL-10, as shown in Figure 2(c), which adds more evidence of the anti-inflammatory effect of PCEE in the acute phase response to CCl_4_. The upregulation of IL-10 by PCEE indicates its ability to downregulate the inflammatory cytokine. The hepatic level of iNOS-2 on mRNA expression in CCl_4_-treated rats was approximately 7.3-fold higher than that in the control rats. There is a dose dependent significant downregulation in rat groups previously treated with PCEE, silymarin + CCl_4_ (Figure 2(d)). This further indicates that PCEE and silymarin have anti-inflammatory activities.

The extent of heme catabolism, as shown in Table 2, illustrated significantly higher levels of bilirubin in serum of CCl_4_-treated rats compared to that of the control group. The same trend is observed in the inducible HO-1 on mRNA expressions which were significantly increased 7.4-fold (*P* ≤ 0.01) in CCl_4_ group compared to control (Figure 2(e)). Conversely, control did not exert any significant changes in HO-1 on mRNA expressions. PCEE and silymarin significantly (*P* ≤ 0.01) decreased the levels of HO-1 on mRNA expression by 5.06-, 2.7-, and 1.6-fold in groups III, IV, and V, respectively.

### 4.7. Histopathological Observations

**Figure 3:**
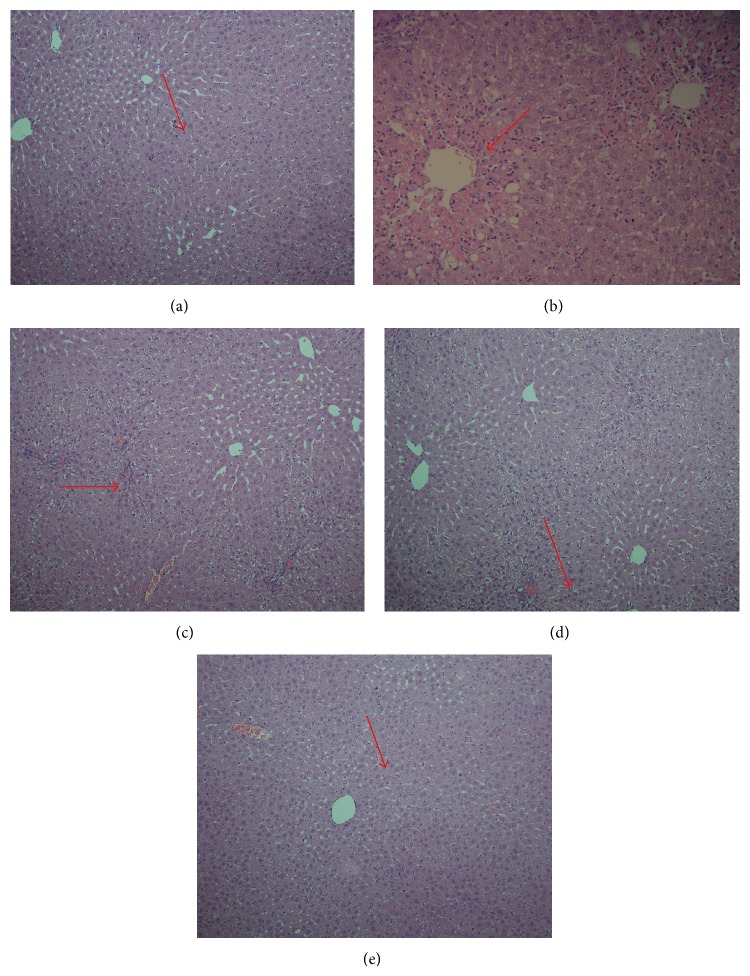
Histopathology of liver tissues. (a) Liver section of normal control rat shows central vein surrounded by hepatic cord of cells (normal architecture), (b) liver section of CCl_4_-treated rats showing massive fatty changes, focal central vein congestion (indicated by arrow), ballooning formation, necrosis with inflammation, and loss of cellular boundaries, (c) liver section of rats treated with CCl_4_ and 250 mg/kg of PCEE showing mild central vein congestion (indicated by arrow), ballooning, and necrosis with sinusoidal dilatation, (d) liver section of rats treated with CCl_4_ and 500 mg/kg of PCEE showing absence of ballooning, inflammatory cells, and regeneration of hepatocytes around central vein toward near normal liver architecture but slight congestion in central vein (indicated by arrow), and (e) liver section of rats treated with CCl_4_ and 10 mg/kg of silymarin showing normal liver architecture.

The histological observations of liver tissues support the results obtained from serum enzyme assays. Liver section of normal control rat shows central vein surrounded by hepatic cord of cells while liver section of CCl_4_-treated rats shows massive fatty changes, focal central vein congestion, vacuolization, necrosis with inflammation, and loss of cellular boundaries. Liver section of rats treated with CCl_4_ and pretreated with PCEE 250 mg/kg shows mild central vein congestion, vocalization, and necrosis with sinusoidal dilatation. Liver sections of rats treated with CCl_4_ and 500 mg/kg of PCEE show absence of vocalization, inflammatory cells, and regeneration of hepatocytes around central vein but slight congestion in central vein and almost toward near normal liver architecture and possessing higher hepatoprotective action. The effect of silymarin was similar to that of PCEE (500 mg/kg) (Figures 3(a)–3(e)).

### 4.8. *In Vitro* Antioxidant Activity of PCEE

The potential antioxidant activity of the PCEE was investigated on the basis of DPPH radical scavenging activity and of inhibition of linoleic acid oxidation. As demonstrated in Table 3, PCEE was able to reduce the stable free radical DPPH to the yellow-colored DPPH at high concentrations (500 and 1000 *μ*g/mL). In addition to that, in the *β*-carotene/linoleic acid model system, the PCEE was also able to inhibit the discoloration of *β*-carotene at a concentration of 1000 *μ*g/mL. The total antioxidant value was 79% (Table 3). The observed antioxidant activity was comparable to that of the positive control, rutin (Table 3).

### 4.9. Phytochemical Screening

The preliminary qualitative screening of PCEE revealed the presence of volatile oils, terpenes, and flavonoids.

## 5. Discussion

The change in dietary habits and chemoprevention show considerable effective strategy against oxidative stress and are the main focus of area of research these days [33]. Various reports have shown that several mutagens and carcinogens cause generation of peroxide radicals, which play a major role in the emergence of cancer and other health disturbances [25, 34]. The current investigation was undertaken to evaluate the possible protective effect of PCEE against carbon tetrachloride induced hepatotoxicity and oxidative stress in rats. CCl_4_ is a known, reliable, and commonly used chemical to induce liver damage. The present study revealed that CCl_4_ induction in rats remarkably increased the level of SGPT, SGOT, GGT, and ALP. CCl_4_ causes acute hepatocyte injuries and altered membrane integrity and as a result enzymes in hepatocytes leak out [35]. However, after pretreatment with PCEE and silymarin, the pathological increases in SGOT, SGPT, *γ*-GGT, and ALP were significantly restored. These results indicate that PCEE and silymarin have the ability to protect against CCl_4_-induced hepatocyte injuries. The mechanism of CCl_4_-induced liver damage is known to be mediated through free radical reactions [10]. The metabolism of CCl_4_ toxicity lies in its biotransformation by the cytochrome P450 system to two reactive metabolites such as trichloromethyl (CCl_3_^+^) free radicals and trichloromethylperoxy (CCl_3_OO^∙^) in the endoplasmic reticulum [36, 37] of the liver and initiated lipid peroxidation process [38].

CCl_4_ is reported to induce hepatic damage as a result of metabolic conversion of the radicals through lipid peroxidation and disturbance of the activities of the antioxidant enzymes [39] and induce oxidative stress and cause liver injury by the formation of free radicals [40]. Carbon tetrachloride, on the other hand, causes noticeable toxicity by enhancing liver lipid peroxidation (LPO), as found by increased concentrations of hepatic malondialdehyde (MDA) [41, 42]. Malondialdehyde, an end product of LPO, in liver tissue serves as an indicator of LPO, which is known to occur in hepatic toxicity due to the generation of reaction oxygen species (ROS) [42]. A significant decrease of MDA level was observed in rats treated with PCEE and silymarin. PCEE might be protecting the hepatocyte by impairing CCl_4_-mediated lipid peroxidation and resulting in the prevention of the generation of free radical derivatives [43]. The liver intoxication provoked by CCl_4_ causes a significant depletion of nonprotein sulfhydryl contents of the liver tissue which is an important indicator towards indicating the oxidative damage of liver. Depletion in NPSH level within living organisms causes tissue injury and further dysfunction [44]. However, pretreatment with PCEE significantly prevented the CCl_4_-induced decreased hepatic NPSH, indicating an antioxidant property of the extract in CCl_4_-induced liver toxicity. These findings show that* Piper cubeba* extract possesses the ability to scavenge reactive free radicals that diminish oxidative stress or damage of the liver tissue and provoke the activities of the hepatic antioxidant enzymes. AS_1_-SH_1_ acts as a nonenzymatic antioxidant, both intra- and extracellularly involved in the protection of normal cell integrity and function by redox and detoxification 15 reaction [45]. Furthermore, the current study manifested a pronounced diminution in liver tissue total protein (TP) level in CCl_4_ only treated rats, whereas TP level in the liver significantly elevated after the administration of PCEE and silymarin. Total protein level can also be used as one of the biomarkers to determine liver function [46]. This indicates that PCEE and silymarin induced hepatic cell proliferation which is sign for liver regeneration.

Catalase (CAT) is an enzymatic antioxidant widely distributed in all animal tissues, and the highest activity is found in the red cells and liver. Serum catalase (CAT) is the most sensitive enzymatic index in liver injury caused by reactive oxygen species (ROS) and oxidative stress [47, 48]. CAT is a hemoprotein which protects the cells from the accumulation of H_2_O_2_ [49]. Therefore, reduction in the activity of CAT may result in a number of deleterious effects due to the assimilation of superoxide radical and hydrogen peroxide. A higher dose (500 mg/kg) increases the level of CAT as produced by silymarin.

To investigate the underlying mechanism, we evaluated the effect of PCEE on the mRNA expression of certain proinflammatory cytokines related inflammation and proliferation. TNF-*α*, IL-6, and IL-10 as acute phase genes are considered to be special biomarkers for inflammation [50]. ROS upregulate NF-*κ*B, which further induced proinflammatory cytokines, such as IL-1*β*, TNF-*α*, and IL-6 [51]. TNF-*α* and IL-6 are key mediators of inflammatory responses and control the expression of the inflammatory gene network. Therefore, the induction of TNF-*α* and IL-6 contributes significantly to the hepatic injury, which is associated with the upregulation of TNF-*α* and IL-6 gene expression that was observed in the CCl_4_ group. This result is therefore in accordance with previous studies [26]. Consequently, the induction of TNF-*α* and IL-6 contributed to the manifestation of the systemic inflammatory response and ultimately to the development of organ failure. The attenuation in level of inflammatory cytokines may explain the accelerated liver regeneration as observed in PCEE and silymarin administrated rat. The release of TNF-*α*, as a proinflammatory mediator in liver apoptosis, is also linked to cytotoxicity induced by CCl_4_ [52, 53].

Interleukin-10 (IL-10) is a major anti-inflammatory cytokine that potently inhibits production of proinflammatory mediators such as TNF-*α* and IL-12 [54]. Treatment of rats with PCEE and silymarin for one week prior to CCl_4_ administration increased the mRNA expression of IL-10 which adds more evidence of the anti-inflammatory effect of PCEE and silymarin in the acute phase response to CCl_4_. The upregulation of IL-10 by PCEE and silymarin indicates its ability to downregulate the inflammatory cytokine. Inflammatory liver involving oxidative stress initiates upregulation of HO-1 mRNA expression and increase in products of heme degradation pathway [55, 56]. Among these products, bilirubin and CO are the key mediators of inducible HO-1 mediated cytoprotection for the reason that they help restore intracellular homeostatic balance under oxidative stress conditions and help suppress inflammation through downregulation of proinflammatory mediators [55, 57–60]. The results of HO-1 on mRNA expression showed that treatment of CCl_4_, indicating the trend of increased HO-1 m RNA expression, while PCEE and silymarin pretreated CCl_4_ rats showed marked reduction in HO-1 on mRNA expression in dose dependent manner. This clearly indicates that PCEE and silymarin have cytoprotective role. The mRNA expressions of iNOS-2 were significantly increased by CCl_4_, which can be attributed to the reported CCl_4_-induced NO production [61, 62]. PCEE pretreatment has a significant reducing effect on iNOS-2 mRNA expression in CCl_4_-treated rats. PCEE and silymarin have mediated cytoprotective actions by suppressing iNOS and HO-1 mRNA expression.

The hepatoprotective effect of the PCEE was further accomplished by the histopathological examinations. PCEE at different dose levels offered hepatoprotection. PCEE 500 mg/kg exhibited similar results to silymarin. The preliminary qualitative analysis indicates the presence of essential oil, terpenoids, and flavonoids which are known antioxidants and anti-inflammatory agents [63, 64].

## 6. Conclusion

Findings of this study demonstrated that PCEE is effective in prevention of CCl_4_-induced hepatic damage in rats. Our results demonstrated that the treatment with PCEE significantly and dose dependently prevented drug induced increase in serum levels of hepatic enzymes. Furthermore, PCEE significantly reduced the lipid peroxidation in the liver tissue and restored activities of defense antioxidant enzymes NP-SH and CAT towards normal levels. The hepatoprotective effect of PCEE is attributed to downregulation of proinflammatory cytokines, for example, TNF-*α* and IL-6 mRNA expression as well as mRNA expression of iNOS and HO-1 gene, and upregulation of the IL-10. Histopathological studies have also shown that the PCEE and silymarin could prevent CCl_4_-induced hepatic damage in the liver.

## Figures and Tables

**Table 1:** List of primers.

Gene	Primer	Reference
TNF-*α*	F-GTAGCCCACGTCGTAGCAAA-3′	[26]
	R-5′-CCCTTCTCCAGCTGGAAGAC-3	

IL-6	F-5-CTTCCAGCCAGTTGCCTTCT	[27]
	GACAG CATTGGAAGTTGGGG	

IL-10	F-5-GGAGTGAAGACCAAAGG-3′	[26]
	R-5′-TCTCCCAGGGAATTCAAATG-3′	

iNoS-2	5′-TTCTTTGCTTCTGTGCTTAATGCG-3	[26]
	5′-GTTGTTGCTGAACTTCCAATCGT-3′	

HO-1	5′-CAGAAGGGTCAGGTGTC-3	[28]
	5′-AGTAACTCCCACCTCGT-3′	

*β*-Actin	5′-CCAGATCATGTTTGAGACCTTCAA-3′	[29]
	5′-GTGGTACGACCAGAGGCATACA-3′	

**Table 2:** Effect of *Piper cubeba* ethanolic extract on liver markers in CCl_4_-induced hepatotoxicity.

Treatments	Dose mg/kg	SGOT (U/L)	SGPT (U/L)	GGT (U/L)	ALP (U/L)	Bilirubin (mg/dL)	LDH (U/L)
Normal		87.28 ± 4.15	31.96 ± 3.05	4.90 ± 0.32	318.50 ± 6.34	0.52 ± 0.02	91.72 ± 3.81
CCl_4_	1.25 mL/kg	222.33 ± 15.57^∗∗∗a^	164.83 ± 9.08^∗∗∗a^	16.18 ± 0.93^∗∗∗a^	471.16 ± 12.03^∗∗∗a^	2.36 ± 0.05^∗∗∗a^	172.68 ± 8.08^∗∗∗a^
PCEE + CCl_4_	250	218.00 ± 6.91^b^	166.16 ± 9.61^b^	14.16 ± 0.41^b^	410.00 ± 8.89^∗∗b^	1.84 ± 0.08^∗∗∗b^	160.34 ± 6.08^b^
PCEE + CCl_4_	500	154.00 ± 6.71^∗∗b^	120.10 ± 8.41^∗∗b^	12.05 ± 0.38^∗∗b^	364.50 ± 12.20^∗∗∗b^	1.53 ± 0.05^∗∗∗b^	139.59 ± 4.21^∗∗b^
Silymarin + CCl_4_	10	137.83 ± 5.85^∗∗∗b^	96.66 ± 5.99^∗∗∗b^	9.33 ± 0.22^∗∗b^	355.50 ± 13.97^∗∗∗b^	0.92 ± 0.05^∗∗∗b^	117.02 ± 3.65^∗∗∗b^

All values represent mean ± SEM. ^**^ *P* < 0.01; ^***^ *P* < 0.001; ^a^ *P* < 0.05; ^b^ *P* < 0.01; ^c^ *P* < 0.001 ANOVA, followed by Dunnett's multiple comparison test.

^∗,∗∗,∗∗∗^Compared to normal group; ^a,b,c^compared to CCl_4_ only group.

**Table 3:** *In vitro* free radical scavenging and antioxidant activity of *Piper cubeba* ethanolic extracts (PCEE).

Plant species	Radical scavenging activity (%)					Total antioxidant activity (%)
	10	50	100	500	1000	1000 (*µ*g/mL)
PCEE	9.8	15.1	33.7	75.2	82.8	79.1 ± 5.26
Ascorbic acid	20.4	71.5	86.9	91.0	93.2	—
Rutin	—	—	—	—	—	92.5 ± 6.54
